# Dissecting the Pathogenesis of Diabetic Retinopathy Based on the Biological ceRNA Network and Genome Variation Disturbance

**DOI:** 10.1155/2021/9833142

**Published:** 2021-10-18

**Authors:** Xiaodan Zhu, Ming Hao, Xinyang Yu, Wenjian Lin, Xuefei Ma, Qian Xu, Lei Cheng, Hongyu Kuang

**Affiliations:** ^1^Department of Endocrinology, The First Affiliated Hospital of Harbin Medical University, Harbin 150081, China; ^2^Department of Anesthesiology, The First Affiliated Hospital of Harbin Medical University, Harbin 150081, China

## Abstract

**Background:**

Diabetic retinopathy (DR) is the most important manifestation of diabetic microangiopathy. It is essential to explore the gene regulatory relationship and genomic variation disturbance of biological networks in DR progression.

**Methods:**

In this study, we constructed a comprehensive lncRNA-mRNA ceRNA network of DR procession (CLMN) and explored its topological characteristics.

**Results:**

Modular and functional analysis indicated that the organization of CLMN performed fundamental and specific functions in diabetes and DR pathology. The differential expression of hub ceRNA nodes and positive correlation reveals the highly connected ceRNA regulation and important roles in the regulating of DR pathology. A large proportion of SNPs in the TFBS, DHS, and enhancer regions of lncRNAs will affect lncRNA transcription and further cause expression variation. Some SNPs were found to disrupt the lncRNA functional elements such as miRNA target binding sites. These results indicate the complex nature of genotypic effects in the disturbing of CLMN and further contribute to gene expression variation and different disease phenotypes.

**Conclusion:**

The identification of individual genomic variations and analysis of biological network disturbance by these genomic variations will help provide more personalized treatment plans and promote the development of precision medicine for DR.

## 1. Introduction

Diabetic retinopathy (DR) is the most important manifestation of diabetic microangiopathy, a common and specific microvascular complication of diabetes, and one of the serious complications of diabetes [1, 2]. Therefore, it is essential to explore the gene regulatory relationship and genomic variation disturbance of biological network in DR progression and further contribute to the prevention and treatment of this complex disease. With the development and improvement of bioinformatics techniques and high throughput RNA sequencing data, we can apply methods such as constructing biological networks and differential expression to analyze the pathogenesis of complex diseases. At the same time, protein-protein interaction (PPI) networks and noncoding RNA-mRNA networks can be constructed for more comprehensive analysis [3]. With the gradual improvement of genomic annotations, multiple sources of disease-related genomic variations such as single nucleotide polymorphisms (SNPs) have been identified, providing opportunities for us to further understand the regulatory mechanisms of complex diseases and discover new therapeutic targets.

Long noncoding RNA (lncRNA) is a new type of noncoding RNA, usually defined as RNA molecules longer than 200 nucleotides [4]. LncRNAs have been shown to be competing endogenous RNAs (ceRNAs) and involved in various biological processes, such as cell growth, antiapoptosis, migration, and invasion [5, 6]. As research progresses, lncRNA has been confirmed to be a key regulator of certain diseases and as an important biomarker in certain biological processes [7]. Experiments confirmed that knockdown of MALAT1 inhibited cell proliferation, migration, and angiogenesis of hRMECs via suppressing the VE-cadherin/*β*-catenin complex through targeting miR-125b. Inhibition of MALAT1 may serve as a potential target for antiangiogenic therapy for DR [8]. HCG18 promotes M1 macrophage polarization through regulating the miR-146a/TRAF6 axis, facilitating the progression of diabetic peripheral neuropathy [9]. HOTTIP improves diabetic retinopathy by regulating the p38-MAPK pathway [10]. Emerging evidence suggests that the expression of lncRNAs can be affected by genomic variations such as SNPs, somatic mutations, and copy number variation [11]. A number of SNPs have been identified in human lncRNA regions and to be associating with various complex diseases including DR [12]. Thus, it is essential to explore the distribution on lncRNAs and perform systematic analysis to evaluate lncRNA-related biological networks affected by genomic variations.

In this study, we have structured our work in several sections. In Materials and Methods, we constructed a comprehensive lncRNA-mRNA ceRNA network (CLMN) related to diabetes and DR. Based on these networks, we performed modular and functional analysis to explore the topological characteristics of lncRNAs and coding genes. In Results, our analysis revealed that the organization of CLMN performed fundamental and specific functions in diabetes and DR pathology. Based on high-throughput RNA sequencing data (GSE102485), we identified differentially expressed hub ceRNA nodes in the CLMN which were important regulators in DR progression. The positive correlation of lncRNA-mRNA relations reveals the highly connected ceRNA regulations and important roles in the regulating of DR pathology. Further, a global map of lncRNA-SNP associations was constructed to show how genomic variants influence biological functions in DR. We explored DR-related SNP and linkage disequilibrium (LD) SNP (*r*^2^ > 0.8) distribution on lncRNAs and found that there was a large proportion of SNPs localized on the functional regions of lncRNAs, such as TFBS, DHS, and enhancer regions, which will affect lncRNA transcription and further cause expression variation. Some SNPs were found to be localized on the functional elements such as miRNA target binding sites of lncRNAs. In Discussion and Conclusions, we concluded that our findings reveal the complex nature of genotypic effects in the disturbing of CLMN and further contribute to gene expression variation and different disease phenotypes. Overall, the identification of individual genomic variations and analysis of biological network disturbance by these genomic variations will help provide more personalized treatment plans and promote the development of precision medicine for DR.

## 2. Materials and Methods

### 2.1. Collection of Diabetes and DR Associated Gene and lncRNAs

We downloaded genes associating with diabetes and diabetic retinopathy (DR) from the DisGeNET (https://www.disgenet.org/) database [13]. A number of 1,349 diabetes genes and 371 DR genes were collected. There were 269 common genes in the intersection of diabetes and DR genes. In addition, to obtain lncRNAs associated with disease genes, we downloaded 2,799 lncRNA-mRNA ceRNA regulations from LncACTdb (2.0) [14]. This ceRNA dataset includes 1,848 lncRNAs and 1,451 protein-coding genes.

### 2.2. Human Protein-Protein Interaction Network

The Human Protein-Protein Interaction (PPI) network was obtained from HuRI (http://www.interactome-atlas.org/) [15]. The network contains 9,064 proteins and 64,006 interactions. We used molecular complex detection (MCODE) to characterize the network. MCODE clusters are generated by MCODE based on topology to identify the function of each highly connected region. Highly interacting nodes in the cluster are identified by maintaining a parameter *K*‐core = 2, a node score cutoff = 0.2, and a maximum depth of 100.

### 2.3. Construction of CLMN

We mapped diabetes- and DR-related genes to the PPI network from HuRI and extracted the largest network component. Further, the lncRNA-mRNA ceRNA regulations from LncACTdb (2.0) were integrated into the PPI network to construct CLMN. The CLMN contains a total of 3,299 nodes and 19,691 edges while lncRNAs, diabetes genes, DR genes, and common genes were illustrated as different color.

### 2.4. High-Throughput RNA Sequencing Data

The expression profile and sample annotation of DR (GSE102485) were downloaded from the GEO database (https://www.ncbi.nlm.nih.gov/geo/). This dataset contains 25 DR and 5 normal retina samples.

### 2.5. Network Illustration and Topological Analysis

The Cytoscape (v3.4.0) software was used to illustrate the ceRNA network. The MCODE plug-in of Cytoscape was used to detect tightly connected modules. The network analyzer plug-in of Cytoscape was used to calculate the topological characteristics of CLMN.

### 2.6. Genomic Variations and Their Association with lncRNAs

We collected DR associating SNPs and LD SNPs (*r*^2^ > 0.8) from the LincSNP 3.0 database [16]. These SNPs were classified into different catalogs according to their relative genomic locations with lncRNAs, including enhancer region, transcription factor binding site, lncRNA region, DNase I hypersensitive sites, open chromatin region, footprint region, and topologically associated domains.

### 2.7. Statistical Analysis

Students' *t*-test was used to measure gene expression variation between DR and normal samples. We used the cutoff of *p* value as 0.05 and the cutoff of fold change as 1.5 as threshold to identify significantly expressed genes. The Pearson correlation analysis was performed to identify coexpressed lncRNA-mRNA gene pairs with *p* value as 0.05. The Mann-Whitney *U*-test was used in comparison of topological characteristics between different types of nodes. All statistical analysis was performed based on the R (3.4.1) software.

## 3. Results

### 3.1. Construction of CLMN and Topological Characteristic Analysis

**Figure 1:**
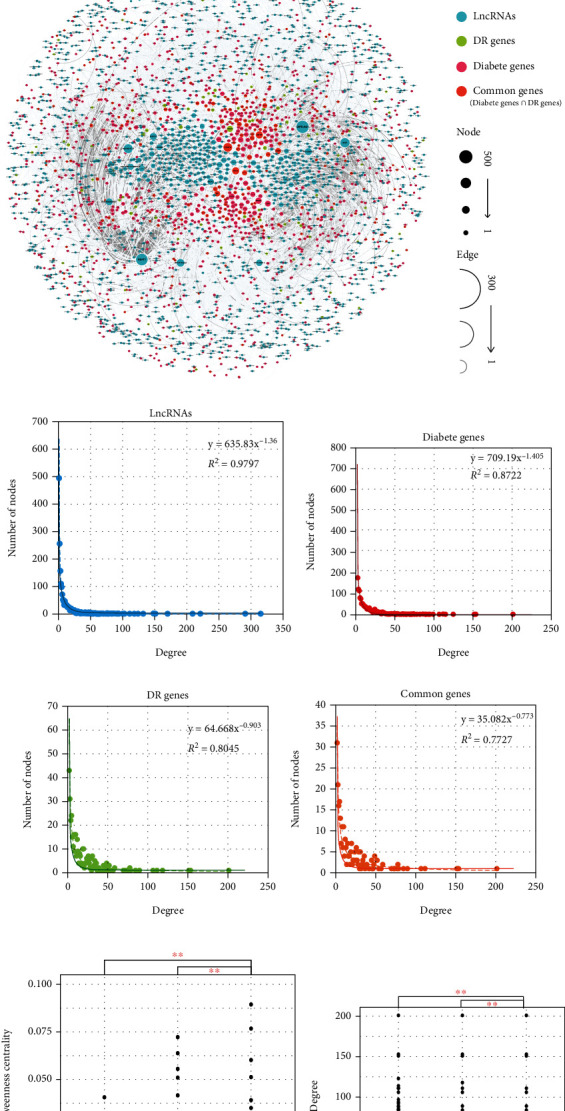
Construction and topological analysis of a comprehensive lncRNA-mRNA network in DR procession. (a) The workflow of lncRNA-mRNA network construction. (b) The global view of CLMN. (c–f) Degree distribution of lncRNAs (c), diabetes (d), DR (e), and common genes (f) of CLMN. (g) Comparison of BC between different types of nodes. (h) Comparison of degree between different types of nodes. (i) Comparison of TC between different types of nodes. ∗∗ indicates statistically significant (*p* < 0.01).

To provide a global view of lncRNA-mRNA regulations in the procession of diabetes and DR, we collected diabetes- and DR-related genes from the DisGeNET [13]. A number of 1,349 diabetes genes and 371 DR genes were collected. There were 269 common genes in the intersection of diabetes and DR genes (Figure 1(a)). We mapped these genes to the PPI network from HuRI and extracted the largest network component. Further, we collected ceRNA regulations consisting of lncRNAs and mRNAs from the LncACTdb (2.0) database [14]. These lncRNA-gene regulations were integrated into the PPI network to construct a comprehensive lncRNA-mRNA network of DR procession (CLMN). The CLMN contains a total of 3,299 nodes and 19,691 edges while lncRNAs, diabetes genes, DR genes, and common genes were illustrated as different color (Figure 1(b)).

We explored the node degree distribution of lncRNAs, diabetes genes, DR genes, and common genes, respectively. Investigation of the degree distribution of lncRNA nodes (*R*^2^ = 0.9797, slope = 1.36), diabetes nodes (*R*^2^ = 0.98, slope = −1.405), DR nodes (*R*^2^ = 0.8045, slope = −0.903), and common nodes (*R*^2^ = 0.7727, slope = −0.773) revealed power-law distributions (Figures 1(c)–1(f)), which indicated that the CLMN was a scale-free network. These results suggested that the CLMN was similar as most biological networks and was well organized by a core set of nodes rather than random networks [6]. Further, we compared several topological properties, including degree, betweenness centrality (BC), and topological coefficient (TC), between different types of nodes. In general, degrees are the number of edges connected to the nodes, while high degree indicates a hub that participated in more regulations. We found that common gene nodes usually had more degrees compared to diabetes nodes and DR nodes (Figure 1(g)). The BC is defined as the ratio of the shortest path between a pair of nodes passing through a given node. Node with high BC reveals a bottleneck that acted as bridges connecting different network modules. Also, we found that common gene nodes had higher BC values compared to diabetes nodes and DR nodes (Figure 1(h)). The TC is calculated to measure the degree to which a node shares links with other nodes in the network. However, the analysis of the results shows that there is no significant difference of TC between the different types of nodes (Figure 1(i)). These observations indicated that the common genes exhibited more specific topological properties and played important roles in the progression of DR.

### 3.2. Modular Organization of CLMN Reveals Fundamental Processes

**Figure 2:**
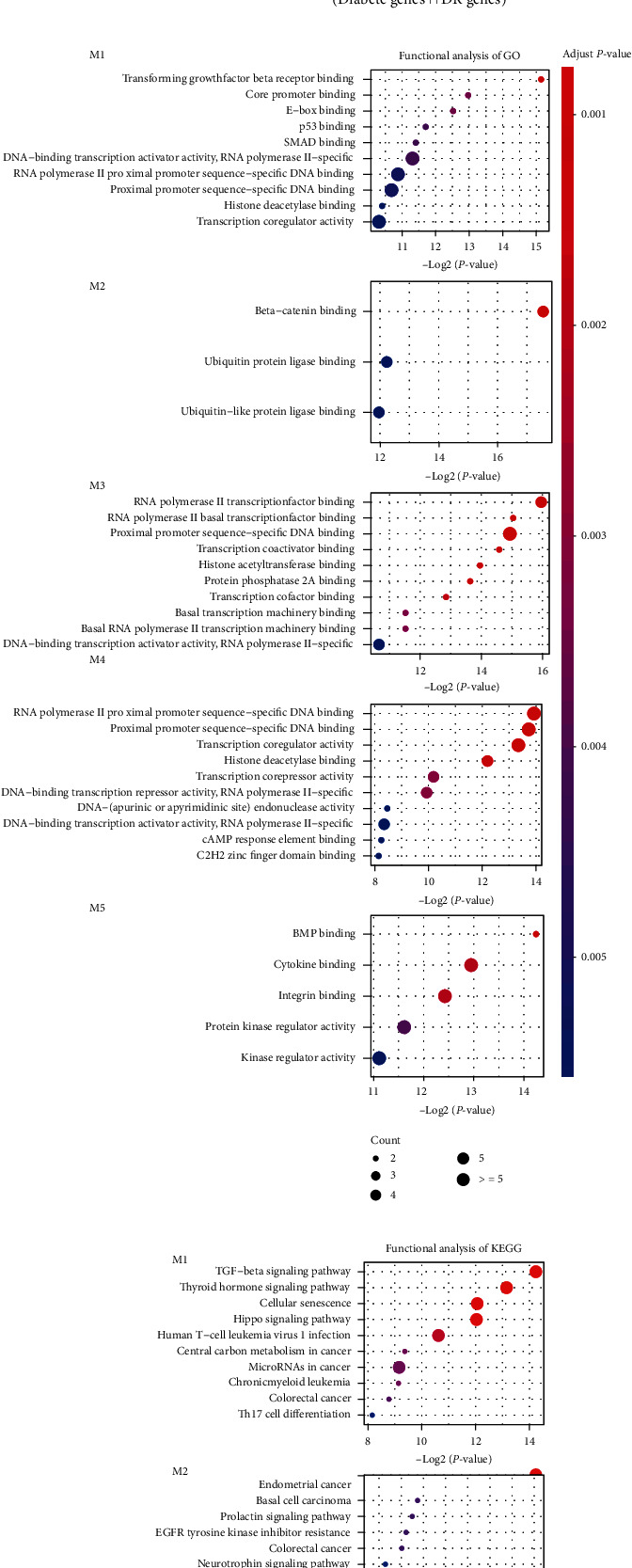
The CLMN modules and functional enrichment analysis. (a) The illustration of five most important modules from CLMN. (b) Functional enrichment analysis of each module based on KEGG and GO background.

Disease-related genes tend to interact with others rather than individual genes and act as regulators of pathological processes. Generally, the protein products encoded by these genes can perform similar functions in the same module. In order to study the common pathology of diabetes and DR, we use the MCODE plug-in in Cytoscape to discover potential modules in the network and select the top 5 modules with the highest complex scores [17] as the important modules in the network. As a result, the five most important modules (with top five highest MCODE score) were identified from the interactive network (Figure 2(a)). Each module contains a subnet of disease-related genes and their ceRNA relationship of lncRNAs. Subsequently, we studied the function of each module by performing functional enrichment analysis of coding genes based on Kyoto Encyclopedia of Genes and Genomes (KEGG) and GO (Gene Ontology) (Figures 2(b) and 2(c)). KEGG is a resource for understanding high-level functions and utilities of the biological pathways that are manually created by capturing knowledge from published literature [18]. GO is a comprehensive system of biological functions, ranging from the molecular to the organism level, across the multiplicity of species in the tree of life [19]. We found that these modules were associating with basic biological processes such as transforming growth factor beta receptor binding, RNA polymerase II transcription factor bindings, and TGF-beta signaling pathway. These are the fundamental processes to maintain the cell growth and transcription of genes of cellular biology. For example, the module M1 was identified to be associating with the TGF-beta signaling pathway, which has diverse effects on cell differentiation, migration, proliferation, and gene expression [20]. This pathway also regulates cell fate during embryonic development and in the maintenance of adult tissue homeostasis [21]. Further, several cancer pathways were found to be associating with these modules indicating there were common genes and regulators beheading complex disease, while dysregulation of these modules can lead to a plethora of developmental disorders and diseases [22]. These results indicated that the organization of CLMN modules performed fundamental processes of cellular biology.

### 3.3. Subnetwork Analysis Elucidates Crucial Functions in DR Pathology

**Figure 3:**
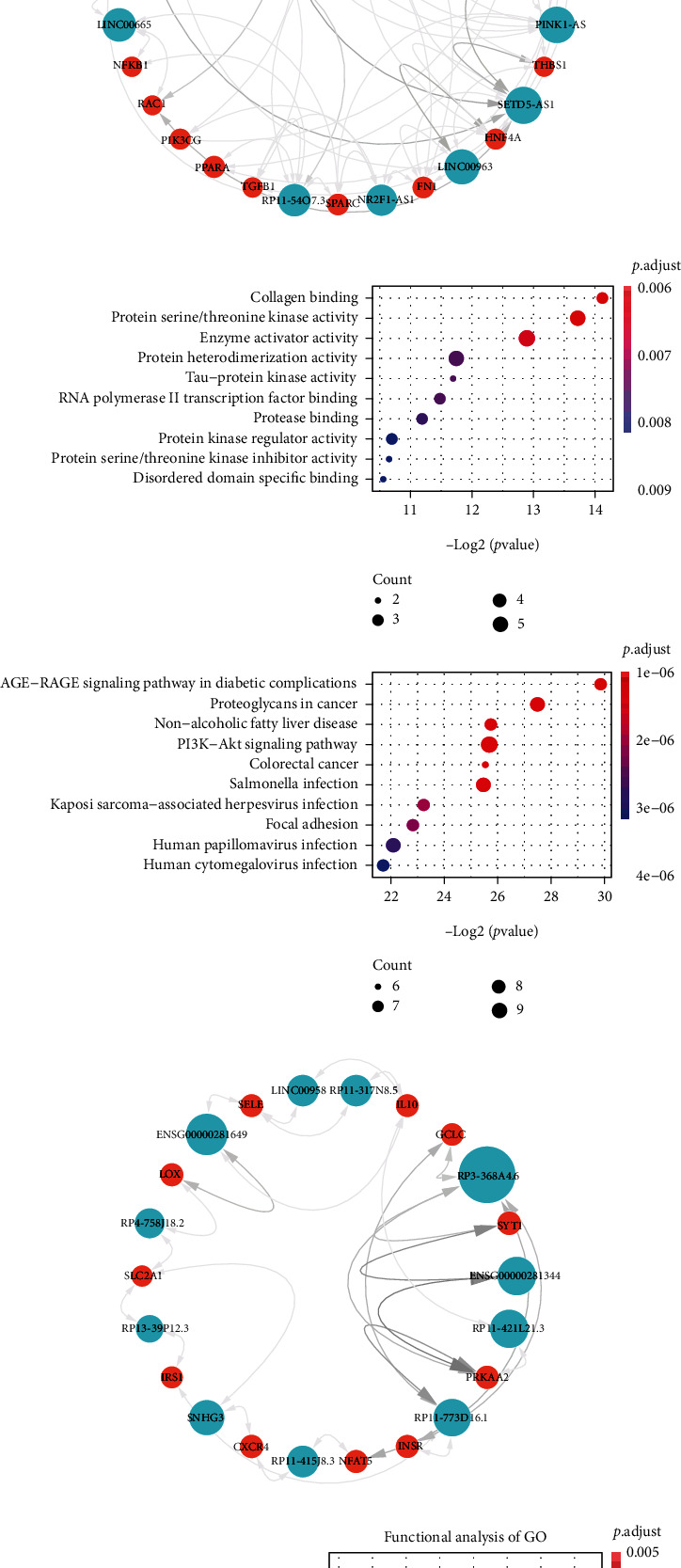
The subnetwork and modules of CLMN consisting of common genes. (a) The Venn diagram of diabetes, DR, and common genes. (b) The subnetwork illustration. This network consisting of 269 common genes and 1,848 lncRNAs. (c–f) Illustration of subnetwork modules and functional enrichment analysis based on KEGG and GO background.

While the CLMN could provide a global view of all possible ceRNA regulations and revealed network modules involved in fundamental processes of cellular biology, the partial subnetworks would indicate a more detailed illustration of ceRNA regulations and functions. Based on the above observations that common genes exhibited more specific topological properties (Figures 1(g)–1(i)), we constructed a ceRNA subnetwork consisting of 269 common genes and 1,848 lncRNAs and further explored modular organization and functional analysis (Figures 3(a) and 3(b)). We used the MCODE plug-in of Cytoscape to find the submodules of this subnetwork. After obtaining the submodules, we performed GO and KEGG analysis on the modules with relatively high scores in the network (Figures 3(c)–3(f)). A module was found to be mainly involved in collagen binding, protein serine/threonine kinase activity, and AGE-RAGE signaling pathway in diabetic complications, which are functions and pathways related to diabetes (Figures 3(c) and 3(d)) [23]. Another module was associating with mTOR signaling and AMPK signaling pathways, which play important roles in the development of diabetes and DR (Figures 3(e) and 3(f)) [24, 25]. Studies have documented the protective effects of the AMPK signaling pathway on DR angiogenesis [26]. Under pathological conditions, AMPK can attenuate angiogenesis by inhibition of mTOR and TGF-*β*/BMP signaling in DR [24]. Insulin resistance and insulin signaling pathways were also found to be associating with this module (Figure 3(f)). In diabetes, insulin resistance will lead to high glucose which can suppress AMPK activity and activate mTOR in procession [27]. These results indicated that the subnetwork consisting of common genes elucidates crucial and specific functions in diabetes and DR pathology.

### 3.4. Identification of Key ceRNA Relations and Functional Analysis

**Figure 4:**
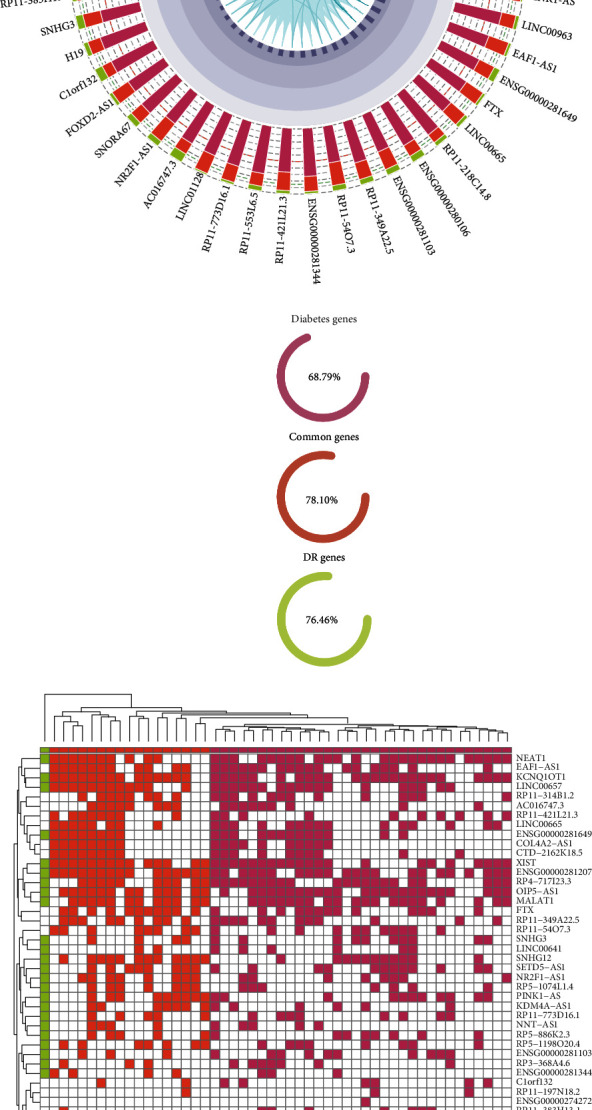
Topological characteristics and functional analysis of key ceRNAs in DR progression. (a) The degree and BC distribution of mRNAs in the CLMN. (b) The degree and BC distribution of lncRNAs in the CLMN. (c) Comparison of node degree between all coding genes and lncRNAs in the CLMN. (d) Comparison of node degree between the hub coding genes and lncRNAs in the CLMN. (e) Comparison of BC between all coding genes and lncRNAs in the CLMN. (f) Comparison of BC between the hub coding genes and lncRNAs in the CLMN. (g) The regulating tendency of hub lncRNAs in regulating diabetes genes, DR genes, and common genes. (h) The regulating proportion of hub lncRNAs in regulating different genes. (i) The ceRNA interaction profile between the top 50 hub lncRNAs and coding genes. (j, k) Functional analysis of hub coding genes based on KEGG and GO.

The regulating effect of a gene can be reflected from topological characteristics based on the background of gene regulatory network [28]. In the CLMN, some well-known coding genes and lncRNAs exhibited higher values of degree and BC, indicating that these nodes participate in more ceRNA regulations and play key roles in DR pathology (Figures 4(a) and 4(b)). For example, the coding genes VEGFA, HGF1R, and BCL2 have been found to be involved in diabetes and DR progression [29–31]. Knockdown of the lncRNA NEAT1 exerts suppressive effects on diabetic retinopathy progression [32]. Another lncRNA XIST regulates hyperglycemia-associated apoptosis and migration in human retinal pigment epithelial cells [33]. Based on the ceRNA theory, lncRNAs are always considered to be upstream of mRNAs and playing driver roles of ceRNA regulation [6]. We compared the topological characteristics between coding genes and lncRNAs in the CLMN (Figures 4(c)–4(f)). In the whole network, coding genes had higher values of degree (Figure 4(c)) and BC (Figure 4(e)). Further, we compared the topological characteristics between hub (defined as nodes with top 50 higher degrees) coding genes and hub lncRNAs and found that lncRNAs exhibited higher values of degree (Figure 4(d)) and BC (Figure 4(f)) than coding genes. To explore the regulating tendency of hub lncRNAs, we calculated their regulating proportion of diabetes genes, DR genes, and common genes regulated by lncRNAs and found that most hub lncRNAs tend to regulate higher proportion of common genes (Figures 4(g) and 4(h)). These results indicate that hub lncRNAs play key roles and control the major component of CLMN.

To illustrate detail ceRNA relationship of hub lncRNAs and coding genes, a ceRNA interaction profile has been built (Figure 4(i)). Among the 50 hub mRNAs, there were 32 diabetes genes, 17 common genes, and 1 DR gene, respectively. Based on hierarchical clustering result, we found that diabetes genes were more likely to be involved in a cluster (with shorter distance than common genes), indicating the stable ceRNA relationship in diabetes. While in the progression of DR, the coding genes were more specifically to be regulated by a certain or a group of lncRNAs. Several well-known DR genes (VEGFA, HGF1R, TIMP3, HIF1A, ATM, BCL2, and STAT3) were coregulated by a panel of lncRNAs (NEAT1, EAF1−AS1, KCNQ1OT1, LINC00657, etc.) indicating the synergistic regulation of these lncRNAs. Functional analysis revealed that the hub coding genes were involved in the AGE-RAGE signaling pathway in diabetic complications, diabetic cardiomyopathy, and several cancer pathways (Figure 4(j)). These coding genes were also associating with response to hypoxia and response to oxygen level based on GO background (Figure 4(k)). The hypoxia process will lead to an increased expression of angiogenic factors and subsequent neovascularisation, which characterize the proliferative phase of DR [34]. These results revealed that hub lncRNAs and coding genes exhibited specific topological characteristics and play key roles in the progression of DR.

### 3.5. Specific Expression Pattern of Hub ceRNA Relations in CLMN

**Figure 5:**
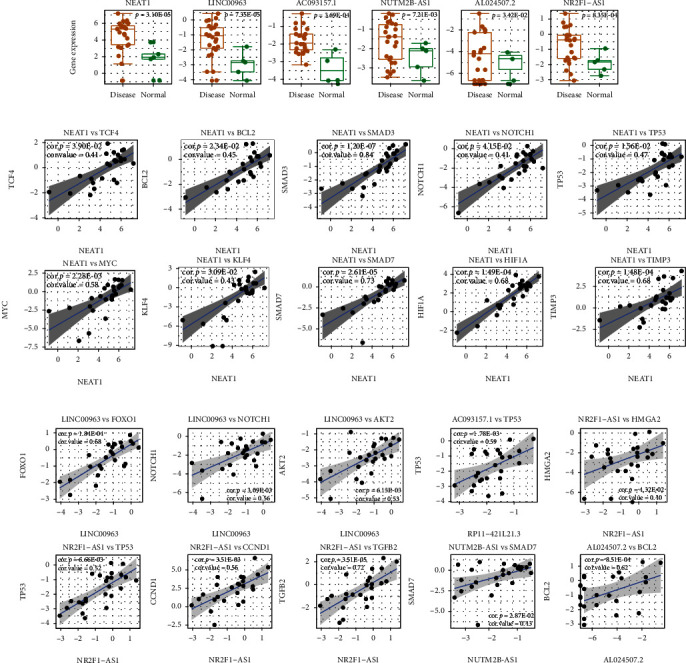
The differential expression and coexpression analysis of hub lncRNAs and coding genes in CLMN. (a) Differentially expressed lncRNAs between DR and normal retina samples. (b) Coexpression patterns of NEAT1 related ceRNAs. (c) Coexpression patterns of other hub ceRNAs. The expression values were log2-transformed.

To characterize the expression patterns of hub ceRNA relations, a high-throughput RNA sequencing dataset from GEO (GSE102485) which contains 25 DR and 5 normal retina samples was used to perform differential expression and coexpression analysis. Several hub lncRNAs have been found to be differentially expressed between DR and normal retina samples (Figure 5(a)). For example, a well-known DR-related lncRNA NEAT1 was highly expressed in DR samples, indicating its risk role in DR progression, which is consistent with previous founding [32]. Some other lncRNAs such as LINC00963, AC093157.1, NR2F1-AS1, NUTM2B-AS1, and AL024507.2 were also upregulated in DR samples. These lncRNAs may be potential risk factors in DR progression. The increased lncRNA expression can enhance downstream coding-gene expression; thus, a functional ceRNA relation can be captured by coexpression analysis [6, 35]. We explored the coexpression patterns of ceRNAs including hub lncRNAs and coding genes (Figures 5(b) and 5(c)). The lncRNA NEAT1 was significantly coexpressed with other hub coding genes (Figure 5(b)). Three of these coding genes (BCL2, HIF1A, and TIMP3) were common genes in diabetes and DR. Positive correlation patterns have also been found in some other ceRNA pairs (Figure 5(c)). Overall, the differential expression of hub ceRNA nodes and positive correlation of their relations reveals the highly connected ceRNA regulations and important roles in the regulating of DR pathology.

### 3.6. A Global Map of Genomic Variations Disturbing CLMN

**Figure 6:**
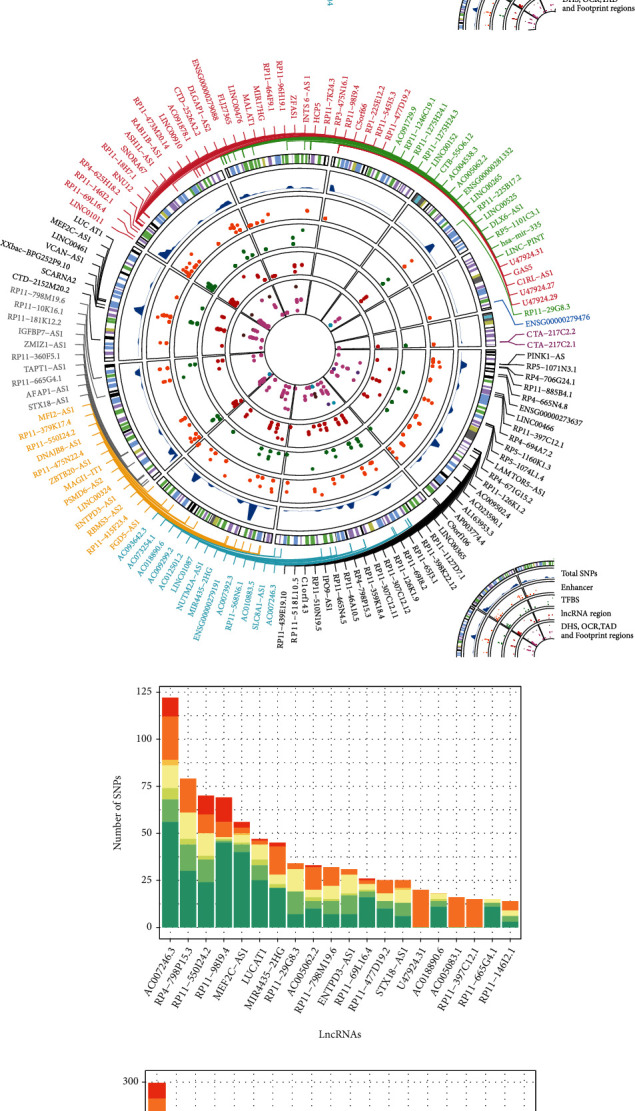
The association between DR-related SNPs and lncRNAs. (a, b) A global map of DR-related SNP and LD SNP distribution and their association with lncRNAs in CLMN. (c, d) Proportion of DR-related SNPs and LD SNPs localized on the different lncRNA regions.

Emerging evidence suggests that the expression of lncRNAs can be affected by genomic variations such as single nucleotide polymorphisms (SNPs), somatic mutations, and copy number variations [11]. A number of SNPs have been identified in human lncRNA regions and to be associating with various complex diseases including DR [12]. To explore the SNP distribution on lncRNAs and their functional affection on the CLMN, we collected DR associating SNPs and LD SNPs (*r*^2^ > 0.8) from the LincSNP 3.0 database [16] and built a global map of lncRNA-SNP associations in DR (Figures 6(a) and 6(b)). These SNPs were classified into different catalogs according to their relative genomic locations with lncRNAs, such as enhancer region, transcription factor binding site, lncRNA region, and DNase I hypersensitive sites. We found that most of the lncRNAs in CLMN were associating with DR SNPs and LD SNPs, indicating the CLMN was under disturbance of genomic variations. There was a large proportion of SNPs localized on the TFBS, DHS, and enhancer regions of lncRNA (Figures 6(c) and 6(d)) which will affect lncRNA transcription and further cause expression variation. Some SNPs were found to be localized on the lncRNA region, which will disrupt the lncRNA functional elements such as miRNA target binding sites.

### 3.7. Dissecting Causative Genomic Variations Affecting Biological Network of DR

**Figure 7:**
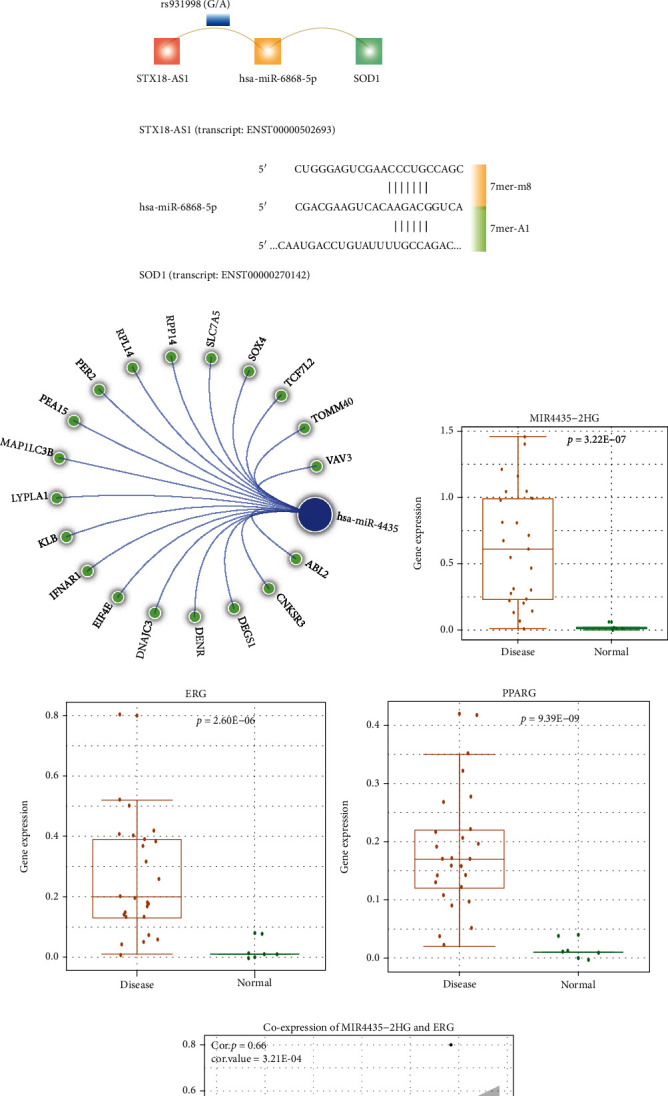
The functional effect of different genomic variations. (a–c) Individuals with different SNP genotypes will gain or lose miRNA binding sites and further disturb a ceRNA relation. (d) Some of the experimentally verified miRNA targets of hsa-miR-4435 were DR-related genes. (e–g) The differential expression of MIR4435-2HG, EGR, and PPARG between DR and normal retina samples. (h) The positive coexpression between MIR4435-2HG and ERG. (i–l) The TFBS binding motifs of EGR and PPARG.

Based on the above global map (Figures 6(a) and 6(b)), we found a SNP rs12108041(C/T) was localized on the lncRNA region of ENTPD3-AS1. According to the ceRNA regulation of CLMN, ENTPD3-AS1 was regulating a DR-related gene SIRT3 by sharing two common miRNA binding sites of hsa-miR-6796-5p and has-miR-1249-5p (Figures 7(a) and 7(b)). SIRT3 has been reported to be involved in regulating neuronal dysfunction of DR [36]. Individuals with the genotype T on ENTPD3-AS1 transcript will generate hsa-miR-6796-5p and has-miR-1249-5p binding sites and construct a ceRNA relation between ENTPD3-AS1 and SIRT3 (Figures 7(a) and 7(b)). Also, the genotype A of rs931998 (G/A) on lncRNA STX1B-AS1 will generate hsa-miR-6868-5p binding sites and further construct a ceRNA relation between STX1B-AS1 and SOD1 (Figure 7(c)). The potential benefit of SOD1 overexpression to inhibit retinal abnormality has been previously studied [37]. The lncRNA MIR4435-2HG, which is a host gene of hsa-miR-4435, has a large proportion of SNPs localized on the TFBS region (Figures 6(c) and 6(d)). We collected experimentally verified miRNA targets of hsa-miR-4435 from miRTarBase (v2020) [38] and found a number of DR-related genes were targeted by hsa-miR-4435 (Figure 7(d)), indicating an important regulating role of MIR4435-2HG in DR progression. By using Chromatin Immunoprecipitation Sequencing (ChIP-seq) data from ENCODE (v112), we found enriched sequencing read peaks of several transcript factors, such as ERG and PPARG, in the TFBS region of MIR4435-2HG (Figure S1). Based on the high-throughput RNA sequencing data (GSE102485), the MIR4435-2HG and two transcript factors were differentially expressed between DR and normal retina samples (Figures 7(e)–7(g)). The lncRNA MIR4435-2HG was significantly coexpressed with ERG (Figure 7(h)). A number of SNPs were found to be localized within the TFBS region of MIR4435-2HG (Figure S1). SNPs localized on the MIR4435-2HG TFBS region will generate or disrupt the binding sites of ERG (Figures 7(i) and 7(j)) and PPARG (Figures 7(k) and 7(l)) bind sites and further affect its transcriptional process. These results indicate the complex nature of genotypic effects in the disturbing of CLMN and further contribute to gene expression variation and different disease phenotypes.

## 4. Discussion

In this study, a comprehensive lncRNA-mRNA ceRNA network of DR procession was constructed to explore the lncRNA regulatory behaviors through ceRNA interactions. At the same time, we divided the CLMN into diabetes-related gene nodes, DR-related gene nodes, and common gene nodes and further analyzed the topological characteristics of the network. Observations indicated that the common genes exhibited more specific topological properties and played important roles in the progression of DR. Modular and functional analysis indicated that the organization of CLMN performed fundamental processes. Modules of CLMN were identified to be involved in basic biological processes such as transforming growth factor beta receptor binding, RNA polymerase II transcription factor bindings, and TGF-beta signaling pathway. These are the fundamental processes to maintain the cell growth and transcription of genes of cellular biology. Further, several cancer pathways were found to be associating with these modules indicating there were common genes and regulators beheading complex disease, while dysregulation of these modules can lead to a plethora of developmental disorders and diseases [22]. Further, we constructed a ceRNA subnetwork consisting of 269 common genes and 1,848 lncRNAs and further explored modular organization and functional analysis. After obtaining the submodules, we performed GO and KEGG analysis and found that the subnetwork consisting of common genes elucidates crucial and specific functions in diabetes and DR pathology. Apart from other techniques, our method provides a global view of all possible ceRNA regulations and revealed network modules involved in fundamental processes of cellular biology. Further, the partial subnetworks would indicate a more detailed illustration of ceRNA regulations and functions of DR. However, our method is mainly performed based on the computational framework and provides a large number of potential regulators associated with DR pathology. Thus, experimental validation is needed to be performed to identify confidential genes in DR.

Based on high-throughput RNA sequencing data, we performed differential expression of hub ceRNA nodes and correlation analysis. Some of the previously reported lncRNAs and ceRNAs were not significantly expressed or coexpressed with ceRNA partners. These may be caused by the individual genomic variations. Emerging evidence suggests that the expression of lncRNAs can be affected by genomic variations such as SNPs, somatic mutations, and copy number variations [11]. To address this issue, we explored SNP distribution on lncRNAs and built a global map of lncRNA-SNP associations in DR. A number of DR-related SNPs and LD SNPs have been identified in human lncRNA regions and to be associating with various complex diseases including DR [12]. To dissect the complex disease pathology from individual genotype to phenotype, systematic analysis was performed to evaluate biological networks affected by genomic variations. We found that there was a large proportion of SNPs localized on the TFBS, DHS, and enhancer regions of lncRNA which will affect lncRNA transcription and further cause expression variation. Some SNPs were found to be localized on the lncRNA region, which will disrupt the lncRNA functional elements such as miRNA target binding sites. These results indicate the complex nature of genotypic effects in the disturbing of CLMN and further contribute to gene expression variation and different disease phenotypes.

## 5. Conclusions

In conclusion, we constructed a comprehensive lncRNA-mRNA ceRNA network of DR procession and performed system analysis of this biological network. Further, we explored the biological networks affected by genomic variations to dissect complex pathology of DR. The comprehensive ceRNA network will provide a global view of all possible lncRNA-mRNA associations under the complex disease background. By exploring the network, some key regulators with specific topological characteristics can be easily identified. For example, the coding genes VEGFA, HGF1R, and BCL2, which exhibiting higher degree and BC in the network, have been found to be involved in diabetes and DR progression [29–31]. Based on the genomic variation, patients can be divided into subsets with distinct phenotypes and outcomes, which allowing specific therapeutic approaches [39, 40]. Thus, mapping of individual genomic variations to genes and analysis of its biological network disturbance by these personalized genomic variations will provide opportunities for us to further understand the regulatory mechanisms of complex diseases and discover new therapeutic targets of DR.

## Data Availability

The data used to support the findings of this study are available from the corresponding author upon request.

## Supplementary Materials

Supplementary Materials Figure S1: enriched sequencing read peaks of EGR1 and PPARG in the TFBS region of MIR4435-2HG based on multiple ChIP-seq datasets. A number of SNPs were found to be localized on the TFBS region of MIR4435-2HG.
