# Supplementary material for: Dissecting the Pathogenesis of Diabetic Retinopathy Based on the Biological ceRNA Network and Genome Variation Disturbance
Source: Comput Math Methods Med. 2021 Oct 18;2021:9833142. doi: 10.1155/2021/9833142 (PMC8545528; doi:10.1155/2021/9833142)
Supplement: Supplementary Materials — Figure S1: enriched sequencing read peaks of EGR1 and PPARG in the TFBS region of MIR4435-2HG based on multiple ChIP-seq datasets. A number of SNPs were found to be localized on the TFBS region of MIR4435-2HG. [file 9833142.f1.docx]

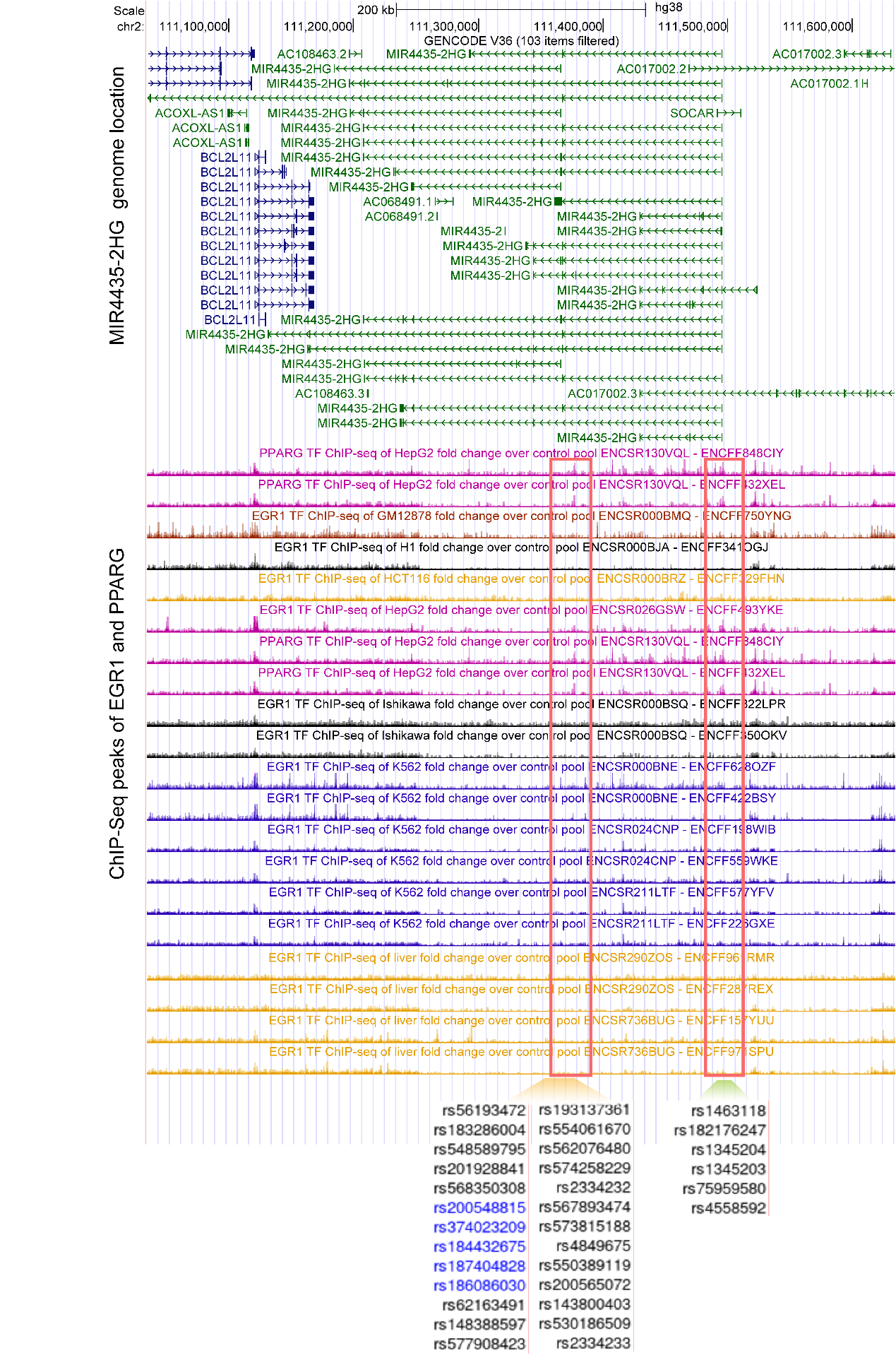
**Figure S1.** Enriched sequencing read peaks of EGR1 and PPARG in the TFBS region of MIR4435-2HG based on multiple ChIP-seq datasets. A number of SNPs were found to be localized on the TFBS region of MIR4435-2HG.
